# Medicaid Payments and Five Star Quality Ratings in the Nursing Home Sector: A Facility Level Analysis

**DOI:** 10.1093/geroni/igaf122.159

**Published:** 2025-12-31

**Authors:** Edward Miller, John Bowblis, Elizabeth Simpson, Marc Cohen

**Affiliations:** University of Massachusetts Boston, Boston, Massachusetts, United States; Miami University, Oxford, Ohio, United States; University of Massachusetts Boston, Boston, Massachusetts, United States; University of Massachusetts Boston, Boston, Massachusetts, United States

## Abstract

Medicaid is the primary and largest single payer for nursing home (NH) care in the United States. The Five-Star Quality Rating System rates NHs from 1 (much below average) to 5 (much above average) based on health inspections, quality measures, and staffing. Ample research on factors that contribute to NH quality exists, yet, due to data constraints, the question of how Medicaid payment levels affect the quality at individual facilities remains largely unexplored. This study examined whether Medicaid payment rates and Medicaid payment-to-cost ratios affect NH 5-star ratings. Star ratings were obtained from Care Compare historical archives, Medicaid per diem payment rates were collected directly from the states, and Medicare payment-to-cost ratios were calculated using estimated Medicaid per diem costs from the Medicare Cost Reports. The study population consisted of 9,475 freestanding NHs in 44 states in 2019. Regression analyses accounted for the clustering of NHs within states and adjusted for known covariates that are associated with NH quality (e.g., ownership status, staffing levels) and state-level fixed effects. Results indicate a positive association between Medicaid payment rates and average star ratings. There is no association between Medicaid payment-to-cost ratios and star ratings when the Medicaid payment-to-cost ratio is below 0.90 (i.e., Medicaid covers less than 90% of estimated cost to care for a Medicaid resident), but a positive association once above 0.90. Policymakers need to consider Medicaid payment as part of NH reform, as higher payment levels that cover a greater proportion of costs are associated with higher quality.

